# Correlation of magnetic resonance images with neuropathology of irreversible metronidazole-induced encephalopathy: an autopsy case report

**DOI:** 10.1186/s12883-022-03006-4

**Published:** 2022-12-15

**Authors:** Yasuo Miki, Yuki Takeuchi, Shingo Murasawa, Shinobu Takayasu, Fumiyasu Tsushima, Shingo Kakeda, Hiroki Mizukami, Koichi Wakabayashi

**Affiliations:** 1 Department of Neuropathology, Hirosaki University Graduate School of Medicine, Hirosaki, 036-8562 Japan; 2 Department of Pathology and Molecular Medicine, Hirosaki University Graduate School of Medicine, Hirosaki, 036-8562 Japan; 3 Department of Endocrinology and Metabolism, Hirosaki University Graduate School of Medicine, Hirosaki, 036-8562 Japan; 4 Department of Radiology, Hirosaki University Graduate School of Medicine, Hirosaki, 036-8562 Japan

**Keywords:** Metronidazole-induced encephalopathy, Magnetic resonance imaging, Diffusion-weighted imaging, Apparent diffusion coefficient, Case report

## Abstract

**Background:**

Neurological symptoms and radiographic abnormalities may remain in a small proportion of patients with metronidazole-induced encephalopathy (MIE). Although experimental animal models of MIE have suggested a Wernicke’s encephalopathy-like pathology, little is known about the histopathological features of MIE. Here we report the first autopsy case of irreversible MIE.

**Case presentation:**

A 72-year-old Japanese woman with pancreatic neuroendocrine tumour and metastatic tumours in the liver developed intraabdominal bleeding from a hepatic abscess. She was administered metronidazole for 79 days (1.5 g/day), which caused dysarthria followed by hand tremor and altered mental status. Brain magnetic resonance imaging at the time of onset revealed hyperintensities in the deep white matter of the bilateral parietal lobes and splenium of the corpus callosum on diffusion-weighted imaging (DWI) with reduced apparent diffusion coefficient (ADC) values. Despite the improvement of dysarthria and hand tremor, her cognition remained affected even after the withdrawal of metronidazole. She died of pancreatic neuroendocrine tumour at the age of 74 years. Histopathological examinations of the brain confirmed a combination of severe demyelination and moderate axonal degeneration, which corresponded to the regions showing abnormal signal intensities on DWI with reduced ADC values. There were no pathological findings suggestive of Wernicke’s encephalopathy in the brain.

**Conclusion:**

We have demonstrated the clinical, radiographic and histopathological aspects of irreversible MIE. Hyperintensities on DWI with reduced ADC values in affected regions may indicate a poor clinical prognosis due to irreversible pathological damage.

**Supplementary Information:**

The online version contains supplementary material available at 10.1186/s12883-022-03006-4.

## Background

Metronidazole is one of the mainstay antibiotics used for anaerobic infections, exhibiting excellent penetration into tissues including the central nervous system (CNS), abscesses, bile and peritoneal fluid [1]. Although well tolerated, metronidazole can be associated with a serious adverse effect on the CNS known as metronidazole-induced encephalopathy (MIE), especially when patients with pre-existing liver disease receive prolonged therapy or large doses of metronidazole [2]. Patients with MIE may develop various types of neurological symptoms or signs including dysarthria (63%), gait instability (55%), limb dyscoordination (53%) and altered mental status (41%) [2, 3]. Magnetic resonance imaging (MRI) of the brain shows characteristic symmetrical hyperintensity in the dentate nuclei (90%) and corpus callosum (44%) on T2-weighted images or fluid-attenuated inversion recovery (FLAIR) [2, 3]. T2 hyperintense lesions in the splenium of the corpus callosum can be found in many conditions [e.g. epilepsy, acute infectious encephalitis, demyelinating diseases, osmotic myelinolysis and acute toxic encephalopathy (MIE)]. Conversely, those in the bilateral dentate nuclei are of great diagnostic value because they are seen in only a few diseases including methyl bromide intoxication, maple syrup urine disease and enteroviral encephalomyelitis in addition to MIE [3]. While these clinical and radiographic features are reversible in most patients with MIE after the withdrawal of metronidazole, a small proportion of patients with MIE can still have residual neurological symptoms and radiographic abnormalities [2]. Several research groups have reported that metronidazole can also cause polyneuropathy due to a combination of demyelination and axonal degeneration of peripheral nerves, with a predilection for large sensory fibers [4, 5]. However, the pathological signature of irreversible MIE remains unknown. Here we report the clinical, radiographical and histopathological features of MIE in a 74-year-old Japanese woman who developed the condition during the course of pancreatic neuroendocrine tumour (P-NET) with metastatic tumours in the liver.

## Case presentation

**Fig. 1:**
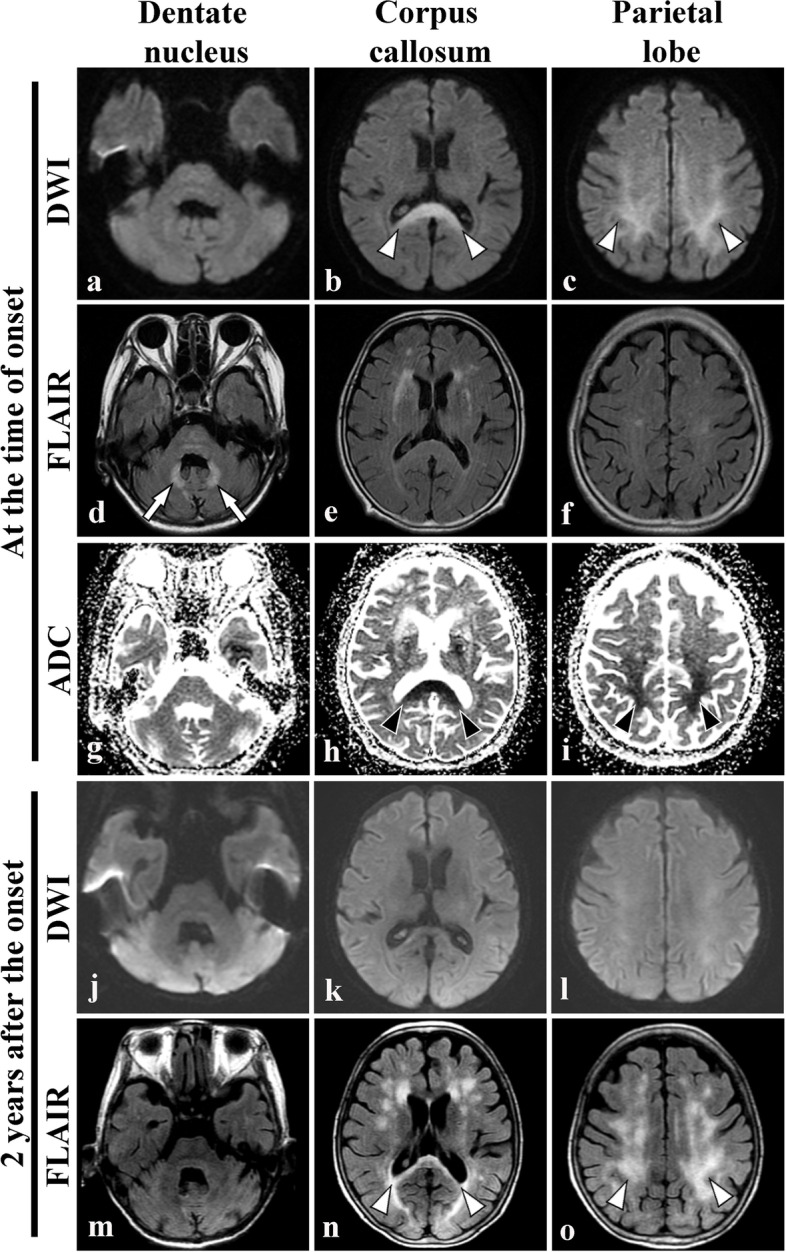
Brain magnetic resonance imaging (MRI) of the present patient with irreversible metronidazole-induced encephalopathy (MIE). **a**-**c** Diffusion-weighted imaging (DWI) at the time of onset shows hyperintensities in the splenium of the corpus callosum (white arrowheads, **b**) and the deep white matter of the bilateral parietal lobes (white arrowheads, **c**). **d**-**f** Fluid-attenuated inversion recovery (FLAIR) imaging of the brain shows hyperintensities in the dentate nuclei (white arrows, **d**), despite a lack of signal abnormalities in the corpus callosum and parietal deep white matter (**e**, **f**). **g**-**i** Apparent diffusion coefficient (ADC) values are reduced in the parietal lobes and splenium of the corpus callosum (arrowheads). Follow-up MRI of the brain 2 years after metronidazole withdrawal reveals near-complete resolution of abnormal intensities on DWI (**j**-**l**). **m** The abnormal signal intensities in the dentate nuclei have also disappeared. (**n**, **o**) However, widespread hyperintensities with diffuse brain atrophy are evident in the splenium of the corpus callosum and deep white matter including the parietal lobes on FLAIR (white arrowheads)

**Fig. 2:**
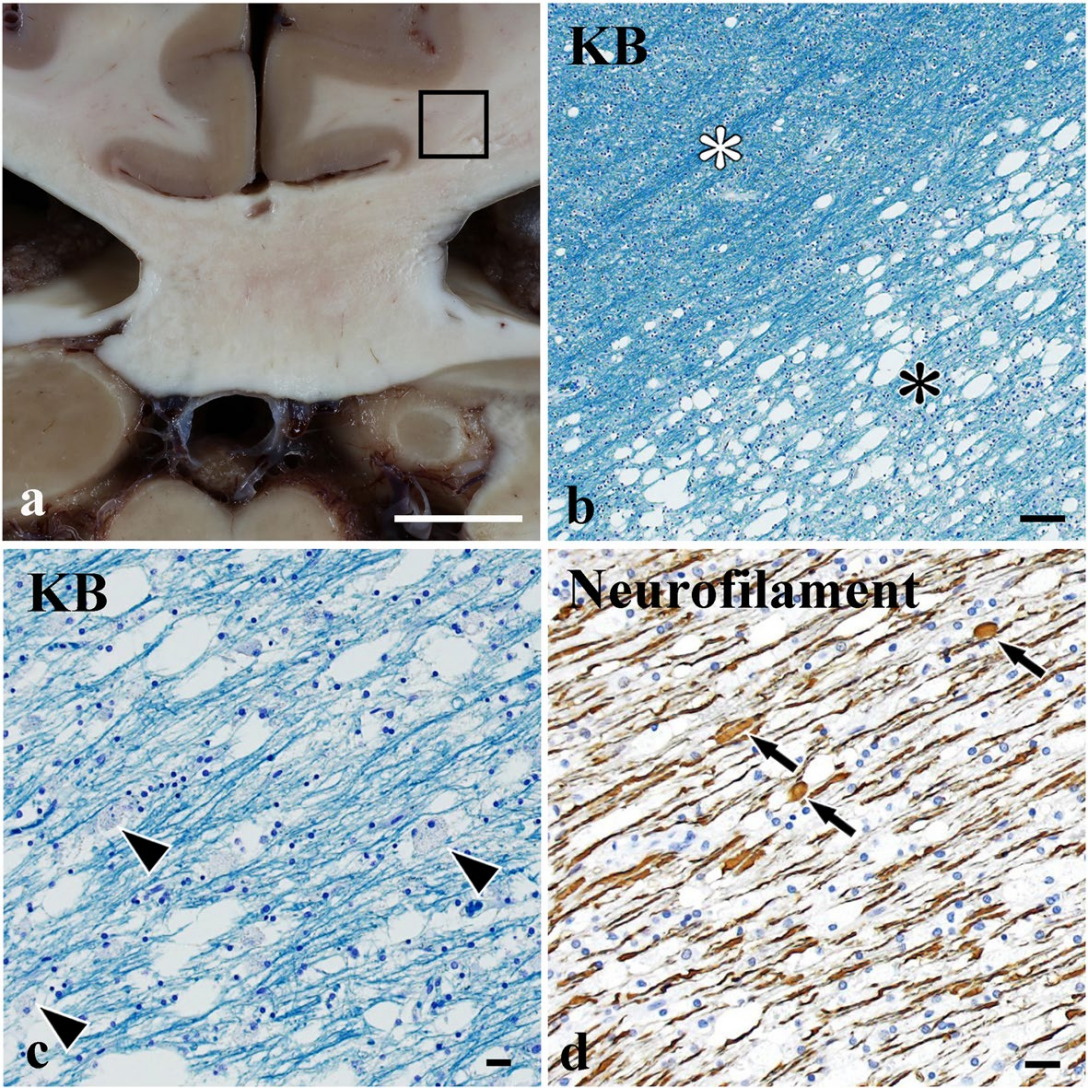
Histopathological features of irreversible MIE. **a **A coronal section of the splenium shows brownish discoloration. **b** Klüver-Barrera (KB) staining in the rectangle in (**a**) reveals severe demyelination in the splenium of the corpus callosum (asterisk), in contrast to the cingulum (white asterisk). Severe demyelination with foamy macrophages (arrowheads, **c**) and moderate loss of axons with swelling (arrows, **d**). Bars = 1 cm (**a**); 100 μm (**b**); 20 μm (**c**, **d**). (**b**, **c**) KB staining; (**d**) immunohistochemistry with anti-neurofilament antibody

A Japanese woman with no clinical history of methyl bromide intoxication, maple syrup urine disease and cognitive impairment was diagnosed as having P-NET and underwent total pancreatectomy with splenectomy and partial resection of the liver (S6) at the age of 70 years. She developed liver metastases six months after the surgery and was administered treatments for P-NET [a synthetic analogue of somatostatin (lanreotide), an inhibitor of mammalian target of rapamycin (everolimus), and a multi-targeted receptor tyrosine kinase inhibitor (sunitinib)]. At the age of 72 years, she developed a hepatic abscess 20 mm in diameter, which caused intraabdominal bleeding. Although we performed abscess drainage, there was a residual abscess which was treated medically. Thus, we administered metronidazole (1.5 g/day) in combination with tazobactam/piperacillin or cefmetazole. However, after 79 days of metronidazole treatment (cumulative dose 118.5 g), the patient developed transient dysarthria followed by hand tremor and altered mental status [mini-mental state examination (MMSE) score 11/30]. Blood examinations showed normal liver function at the time of onset. Brain MRI at the time of onset demonstrated hyperintensities in the deep white matter of the bilateral parietal lobes and splenium of the corpus callosum on diffusion-weighted imaging (DWI), and in the dentate nuclei on FLAIR images (Fig. 1a-f). The apparent diffusion coefficient (ADC) was reduced in the corresponding regions of the parietal lobes and corpus callosum, but not in the dentate nuclei (Fig. 1 g-i). Withdrawal of metronidazole led to improvement of the hand tremor, and resolution of hyperintensity in the dentate nuclei on FLAIR, thus allowing a diagnosis of MIE to be made. Although the blood thiamine level was within normal limits (27 ng/mL), we also administered thiamine intravenously. Despite these treatments, her cognition remained affected 6 and 12 weeks after drug withdrawal (MMSE score 19/30 and 21/30, respectively, the former of which showed severe impairment of orientation and attention). Follow-up MRI of the brain after two years demonstrated widespread hyperintensities in the deep white matter including the parietal lobes and the splenium of the corpus callosum on FLAIR (Fig. 1j-o). She died of P-NET at the age of 74 years. Histopathologic examination of the liver showed that the tumour cells had a diffuse alveolar pattern with a macrovascular stroma, being immunoreactive for chromogranin A and synaptophysin, thus allowing a pathological diagnosis of NET G3 (KI-67 > 20%). Klüver-Barrera staining of the brain revealed severe demyelination with foamy macrophages in the splenium of the corpus callosum and deep white matter of the parietal lobes (Fig. 2a-c). Immunohistochemistry with an antibody against neurofilament (clone 2F11; Dako Cytomation, Glostrup, Denmark; 1:200) also confirmed moderate axonal loss with axonal swellings in the same region (Fig. 2d). On the other hand, minute loss of neurons in the dentate nuclei was noted with preservation of the superior cerebellar peduncles (data not shown). There were no tumour cells, inflammatory cell infiltration suggestive of viral encephalomyelitis, petechial haemorrhages or haemosiderin-laden macrophages in the brain.

## Discussion and conclusions

To our knowledge, this is the first autopsy case of irreversible MIE to have been reported. In our patient, severe demyelination with moderate axonal degeneration in the affected regions was a cardinal feature, similar to that in metronidazole-induced peripheral neuropathy [4, 5]. Interestingly, the regions showing hyperintensities on DWI with reduced ADC values corresponded to those with demyelination and axonal degeneration. By contrast, the abnormal signal intensity of the dentate nuclei disappeared after metronidazole withdrawal, and accordingly the pathological features in the dentate nuclei were largely resolved. These radiographic and pathological features may well explain the persistent cognitive impairment even after drug withdrawal, in contrast to the improvement of hand tremor.

Sørensen et al. performed a systematic literature review of 112 papers, which comprise 136 patients with MIE. They reported cumulative dose of metronidazole in 110 MIE cases available (a lower quartile of 36 g, a median of 65.4 g and upper quartile 110.8 g) and total duration of treatment in 125 cases (a lower quartile of 19.5 days, a median of 35 days and upper quartile of 63 days) [2]. They further examined these factors in six MIE cases with severe residual neurological symptoms [2]. Interestingly, similarly to our patient, all of them showed abnormal signal intensity in the cerebral white matter on T2/FLAIR, DWI or ADC. However, duration of treatment and cumulative dose of metronidazole varied among cases (duration of treatment: 6 to 180 days; cumulative dose: 33 to 250 g) [2]. Although these factors in our patient were beyond the upper quartiles, the findings in six patients with residual neurological symptoms also suggest some additional factors associated with the occurrence of MIE.

The mechanism of metronidazole-induced neurotoxicity has been unclear. In an early study reported by Rogulja et al., a CNS Wernicke-like picture was found in rats treated with metronidazole (800 mg/kg/day) [6]. Recently, Hassan et al. have shown that metronidazole causes thiamine deficiency and oxidative stress in rats; haematoxylin and eosin staining revealed some degeneration of Purkinje cells in the cerebellum [7]. These findings suggest an association of metronidazole with the metabolism of thiamine in experimental models of MIE. On the other hand, acute Wernicke’s encephalopathy in the human brain is characterized by the presence of petechial haemorrhages in subcortical regions around the third and fourth ventricles. In the chronic stage, rarefaction of the mammillary bodies with haemosiderin-laden macrophages is commonly observed [8]. In our patient, however, neither extravasation of erythrocytes nor haemosiderin-laden macrophages was evident in the affected regions, indicating some differences in the mechanism of MIE between humans and experimental models. In MRI of the brain, callosal and white matter lesions showed diffusion restriction. Thus, cytotoxic oedema may well be triggered by the toxic effect of metronidazole, although pathological examinations could not identify the direct cause of demyelination and axonal loss.

In conclusion, we have reported the clinical, radiographic and pathological features of irreversible MIE. Hyperintensities on brain DWI with reduced ADC values in patients with MIE may indicate an irreversible pathological change involving severe demyelination and axonal degeneration, and therefore a poor clinical prognosis.

## Supplementary Information

**Additional file 1.**

## Data Availability

All data to support the findings in the present study are included in the manuscript.

## Abbreviations

ADC: Apparent diffusion coefficient
CNS: Central nervous system
DWI: Diffusion-weighted imaging
FLAIR: Fluid-attenuated inversion recovery
MIE: Metronidazole-induced encephalopathy
MRI: Magnetic resonance imaging
P-NET: Pancreatic neuroendocrine tumour
