# Supplementary material for: Correlation of magnetic resonance images with neuropathology of irreversible metronidazole-induced encephalopathy: an autopsy case report
Source: BMC Neurol. 2022 Dec 15;22:485. doi: 10.1186/s12883-022-03006-4 (PMC9753291; doi:10.1186/s12883-022-03006-4)
Supplement: Supplementary file 1 — Additional file 1. [file 12883_2022_3006_MOESM1_ESM.pdf]

CARE case report guidelines

CARE Checklist of information to include when writing a case report

(cc) BY-NC-ND

Topic	Item	Checklist item description	Reported on Line
Title	1	The diagnosis or intervention of primary focus followed by the words "case report"	Page1, line3
Key Words	2	2 to 5 key words that identify diagnoses or interventions in this case report, including "case report"	Pge 2, line11-12
Abstract (no references)	3a	Introduction: What is unique about this case and what does it add to the scientific literature?	page 4, line18
	3b	Main symptoms and/or important clinical findings	page5, line6-16
	3c	The main diagnoses, therapeutic interventions, and outcomes	page6, line 5
	3d	Conclusion—What is the main "take-away" lesson(s) from this case?	page8, line12-15
Introduction	4	One or two paragraphs summarizing why this case is unique (**may include references**)	page8, line12-15
Patient Information	5a	De-identified patient specific information.	page 5-7
	5b	Primary concerns and symptoms of the patient.	page 4-5
	5c	Medical, family, and psycho-social history including relevant genetic information	page 5, line6
	5d	Relevant past interventions with outcomes	page 5, line7-8
Clinical Findings	6	Describe significant physical examination (PE) and important clinical findings.	page 5, line 15-16
Timeline	7	Historical and current information from this episode of care organized as a timeline	page5-page6
Diagnostic Assessment	8a	Diagnostic testing (such as PE, laboratory testing, imaging, surveys).	page6 line3-10
	8b	Diagnostic challenges (such as access to testing, financial, or cultural)	page4, line 12-14
	8c	Diagnosis (including other diagnoses considered)	page 6, line5
	8d	Prognosis (such as staging in oncology) where applicable	page6, line10
Therapeutic Intervention	9a	Types of therapeutic intervention (such as pharmacologic, surgical, preventive, self-care)	page6, line3-4
	9b	Administration of therapeutic intervention (such as dosage, strength, duration)	page6,line3-4
	9c	Changes in therapeutic intervention (with rationale)	page6,lin3-4
Follow-up and Outcomes	10a	Clinician and patient-assessed outcomes (if available)	page6,line3-4
	10b	Important follow-up diagnostic and other test results	page6,line8-9
	10c	Intervention adherence and tolerability (How was this assessed?)	page6,line3-4
	10d	Adverse and unanticipated events	N/A
Discussion	11a	A scientific discussion of the strengths AND limitations associated with this case report	page7-8
	11b	Discussion of the relevant medical literature **with references**.	page7-8
	11c	The scientific rationale for any conclusions (including assessment of possible causes)	page7-8
	11d	The primary "take-away" lessons of this case report (without references) in a one paragraph conclusion	page8, line12-15
Patient Perspective	12	The patient should share their perspective in one to two paragraphs on the treatment(s) they received	N/A
Informed Consent	13	Did the patient give informed consent? Please provide if requested	Yes ☑ No ☐
